# Health Literacy in the Context of Implant Care—Perspectives of (Prospective) Implant Wearers on Individual and Organisational Factors

**DOI:** 10.3390/ijerph19126975

**Published:** 2022-06-07

**Authors:** Constanze Hübner, Mariya Lorke, Annika Buchholz, Stefanie Frech, Laura Harzheim, Sabine Schulz, Saskia Jünger, Christiane Woopen

**Affiliations:** 1 Cologne Center for Ethics, Rights, Economics, and Social Sciences of Health (CERES), University of Cologne and University Hospital of Cologne, Universitätsstraße 91, 50931 Cologne, Germany; laura.harzheim@uni-koeln.de (L.H.); sabine.schulz@uni-koeln.de (S.S.); 2 Department of Otolaryngology, Hannover Medical School, Carl-Neuberg-Str. 1, 30625 Hannover, Germany; buchholz.annika@mh-hannover.de; 3 Department of Ophthalmology, Rostock University Medical Center, Doberaner Str. 140, 18057 Rostock, Germany; stefanie.frech@med.uni-rostock.de; 4 Department of Community Health, University of Applied Health Sciences Bochum, Gesundheitscampus 6-8, 44801 Bochum, Germany; saskia.juenger@hs-gesundheit.de; 5 Center for Life Ethics, University of Bonn, 53113 Bonn, Germany; chwoopen@uni-bonn.de

**Keywords:** health literacy, decision making, values, implant care, ethical aspects, health-literacy development, cochlear implants, glaucoma implants, cardiovascular implants

## Abstract

The continuous development of medical implants offers various benefits for persons with chronic conditions but also challenges an individual’s, and the healthcare system’s, ability to deal with technical innovation. Accessing and understanding new information, navigating healthcare, and appraising the role of the implant in body perceptions and everyday life requires health literacy (HL) of those affected as well as an HL-responsive healthcare system. The interconnectedness of these aspects to ethically relevant values such as health, dependence, responsibility and self-determination reinforces the need to address HL in implant care. Following a qualitative approach, we conducted group discussions and a diary study among wearers of a cochlear, glaucoma or cardiovascular implant (or their parents). Data were analysed using the documentary method and grounded theory. The data reveal the perceptions of implant wearers regarding the implant on (1) the ability to handle technical and ambiguous information; (2) dependence and responsibility within the healthcare system; and (3) the ethical aspects of HL. Knowing more about the experiences and values of implant wearers is highly beneficial to develop HL from an ethical perspective. Respective interventions need to initially address ethically relevant values in counselling processes and implant care.

## 1. Introduction

The continuous development of implantable technologies offers various benefits for persons with chronic conditions, but also challenges the ability of those affected, their doctors, and the healthcare system, to deal with technical innovation. The integration of technical devices in the human body directly intermingles with individual and social values such as health, (in)dependence, responsibility and self-determination. Furthermore, implant wearers need to develop technical and health competences to keep up with a significant amount of fast-changing technical and health information. The actors involved in implant care also face the challenges of reducing barriers to information, communication and navigation for their clients. In addition, those actors may support implant wearers in their efforts to increase their quality of life *via* and *despite* constant technical upgrades and accompanying uncertainty.

Implant wearers suffer chronic conditions and often have long-term experiences with the healthcare system. Such patients may be savvy with terminology that is relevant to their conditions, but might have difficulties in other fields, such as risk communication or the appraisal of statistical information [1,2,3]. With respect to implant care, the knowledge about the chronic condition pairs with understanding technology- and implant-related information. Herein, two levels of health literacy (HL) become particularly relevant. On the one hand, (1) the individual level regarding competencies of handling and appraising technical and medical information as well as communication skills to engage in informed decision making has to be considered. On the other hand, (2) the organisational level with respect to the responsivity of the healthcare system to individuals’ information needs with regard to their moral values and convictions is of relevance. These aspects underline the essential role of HL, referred to as the capacity of individuals to handle health- and implant-related information (individual HL) [4] and as the responsivity of the healthcare system to individuals’ information needs (organisational HL) [5]. This study provides unique insights on HL in the context of implant care, since, to our knowledge, this topic has not been researched yet. Both individual and organisational HL are described in more detail in the following section.

In the individual lifeworld, wearing an implant has its medical side, where individual HL plays a significant role for organising everyday life with the implant and managing the chronic health condition. In this case, individual HL exceeds its functional dimension as the capacity to obtain, process and understand certain health-related information to be able to make appropriate health decisions [6]. It encompasses critical, communicative [7], and navigation- and technology-related HL. Critical HL relates to the critical appraisal of health information [8]; communicative HL is described as “more advanced cognitive and literacy skills, which together with social skills, can be used to actively participate in everyday activities, to extract information and derive meaning from different forms of communication and to apply new information to changing circumstances” [9] (pp. 263–264); navigation HL describes the competences of individuals to orient themselves in healthcare systems [10]; technology-related HL relates to the individual’s ability to handle health-related technical information and to successfully operate technical devices. Organisational HL comprises the responsivity of the healthcare system to the information needs [6] of (prospective) implant wearers. In implant care, it therefore addresses the providers’ responsibility to offer sufficient access to adequate (technical) information and enable the process of information appraisal incorporating the everyday experiences of implant wearers.

Handling medical and technology-related information is essential for decision making and living with an implant. However, as outlined above, in implant care it is also necessary to deal with the image of one’s own body, adjust to the change in everyday habits and reflect on a new kind of dependence. This can be experienced and processed differently and is necessarily connected to ethically relevant values. Following a bio-psychosocial perspective, for example, *health* is not only determined by biomedical factors (“absence of disease”) but also comprises mental and social components [11] which differ interpersonally. Accordingly, navigating within the healthcare system or the acceptance of implants as a treatment option can also vary, depending on the subjective understanding (in the following, prospective implant wearers (glaucoma) and children of parents with cochlear implants are also implied) of health and the individual expectations of body functionality. An implant may enable social participation [3], decelerate disease progression, or compensate for an impairment [12] but also prevents the individual from a sudden death [13]. In this context, values such as *self-determination, dependence and responsibility* play a central role, especially in terms of deciding for or against an implant (or the proxy decision that has to be made for the child) or in cases where a decision against the implant is not an actual option. This also implies that the individual is faced with a fundamentally new degree of *dependence* and *responsibility*, not only in the decision-making process [14], but also in the often lifelong management of the implant and the disease itself. The feeling of responsibility is thereby shaped by individual attitudes and competences (HL). These aspects demonstrate the relevance of social convictions on health and disease and their manifestation with regards to social participation and individual life planning.

The HL competences elaborated above can, in turn, promote ethically relevant values, emphasising the ethical relevance of HL development. This lends legitimacy to approaching HL from an ethical perspective.

Previous research has repeatedly focused on theoretical and conceptual dimensions of HL and its operationalisation [15], as well as different types of HL and empirical data assessing the HL of different populations [16]. There is also research on the ethical dimensions of HL more generally [3,17,18], whereas Watson (2019) [3] offers recommendations for HL development in the context of implant care. Two further studies address empowerment and communicative responsibility [19,20]. Nonetheless, these are not directly related to HL in the context of implant care and ethics, but rather provide an area of analogy for the better contextualisation of this article. The ethical approach to HL and HL development in the field of implant care is new in the existing research landscape. This study adds a further perspective on HL in relation to implantable technology.

Given the complexity and entanglement of implant care, HL, and ethics, it is essential to learn more about the perspectives and values of implant wearers and incorporate their experiences into the research process. This may help identify gaps in the published research and provide information and perspectives on the ethical and social values related to health technologies [21,22,23]. Exploring lived experiences of implant wearers can help to understand how ethical values are reflected in implant care and offer references for ethically meaningful HL development. This study presumes that individual values and social convictions affect individual and organisational HL in implant care. At the same time, HL promotes various ethical values. HL development in the field of implant care is therefore strongly influenced by ethically relevant values and an ethical responsibility itself.

As part of a joint project, this study aims to offer insights into the individual processes of navigating medical and technical information of cochlear-, glaucoma- or cardiovascular-implant wearers as well as decision making in implant care, which is characterised by constant innovations and technical upgrades. Against this background, we sought to shed light on the connection of HL and ethics in implant care and investigate possibilities for HL development. The leading research questions are: (1) What fosters HL development in the context of implant care from an ethical and patient-centred perspective? (2) How can HL initiatives in implant care be enhanced by the insights of implant wearers?

## 2. Materials and Methods

Following a qualitative approach, group discussions as well as diaries constitute appropriate methods to explore individual perspectives and opinions on implant care, shared understandings or controversies, which evolve through a dynamic discussion with others [24], and to capture contextual experiences in a direct and longitudinal manner [25]. This approach is well-suited to identifying different aspects of decision making, experiences in navigating the healthcare system and quality of life, as well as to obtain insights into the everyday lives of implant wearers and the associated aspects of dealing with implants in a wide range of situations (doctor’s visits, check ups, medication, everyday errands, social relationships, everyday activities). Therefore, remunerated group discussions (GDs) (N = 6) and a diary study (DS) (*n* = 13) with individuals wearing cochlear, glaucoma or cardiovascular implants, and parents of children with a cochlear implant, were conducted. Since the data-collection period coincided with the COVID-19 pandemic, both methods had to be adjusted to an online setting. Ethics approval was obtained in November 2020 (Nr: 20-1176_1) by the Medical Faculty of the University of Cologne. According to the research plan, the study had to be conducted in the period between November 2020 and October 2021. The decision for the time span of the study was based on two main factors: (1) based on previous experience, the researchers anticipated a difficult recruiting process in the field of glaucoma and cardiovascular implants and (2) data collection and analysis were performed in an iterative process.

The data were analysed following the documentary method [1] and the principles of grounded theory [26]. For data validation and methodologically well-founded ways of gaining knowledge from different perspectives [27], a triangulation of methods [28] and of researchers [29] was performed.

### 2.1. Recruitment

Participants were recruited in cooperation with the clinical project partners (patient registries of hospitals) or online by contacting organisers of support groups and relevant forums. Participants were selected via purposive sampling based on the eligibility criteria shown in Table 1.

A great number of cochlear-implant wearers (post-lingual) were interested in study participation. In collaboration with the Hannover Medical School, purposive sampling focused on the following participant characteristics to cover the diversity of implant wearers: sex, age, experience with the implant, complications after surgery, and communication skills. The number of (prospective) glaucoma and cardiovascular-implant wearers was manageable, so that all interested persons could participate in the study after having provided written consent.

### 2.2. Group Discussions

The GDs were conducted via *GoToMeeting* in compliance with data protection regulations. In advance, the participants received detailed instructions on using the platform. Technical support via telephone or e-mail was provided before and during the discussion. A team of three researchers was responsible for conducting the GDs: one moderator, technical support and a substitute moderator in case of technical difficulties.

Between December 2020 and April 2021, a total of six GDs with (prospective) implant wearers (or with parents of children wearing a cochlear implant) of cochlear (N = 3), glaucoma (N = 2) and cardiovascular (N = 1) implants were conducted. The number of participants varied within the groups between 2–9 persons. Across all areas, 26 individuals participated in GDs (Table 2).

All GDs were recorded using *GoToMeeting* and audio recording (as a back-up). For method evaluation purposes, participants were provided with an internet link for a brief online questionnaire.

The course of each GD was supported by a power-point presentation, which included introductory slides containing researchers’ affiliations, several communication rules and information about data-protection requirements. In the beginning, participants were asked to freely associate to a list of keywords related to implant ethics and previously identified through literature research (e.g., “quality of life”, “decision making”, “care”) (see, Appendix A, Table A1). The presentation continued with guiding questions regarding disease- or implant-related decision making, handling of information and future prospects. Subsequently, the participants were given the opportunity to comment or address further aspects. The moderators let the conversations run as freely as possible and only intervened when necessary (e.g., for time-management purposes). The aim was to support the natural flow of the conversation and to ensure active participation by everyone [22].

### 2.3. Diary Study

For this study, the DS complemented the data generated via GDs and interviews. The design of the method was developed referring to the checklist by Janssens et al. [21]. The participant information and the supporting materials developed for the DS were based on the preliminary analysis of the GDs (see Appendix B, Table A2). The aim was to shed light on certain aspects, illuminate them in greater depth, and to reveal aspects that had not yet been discussed in the GDs. To increase compliance, participants were offered personalised feedback reports based on their recordings [21]. A total of *n* = 13 individuals were recruited for the DS: seven wearing cochlear, four glaucoma and two cardiovascular implants (see Table 3.). Apart from the written study information, the participants were also introduced (either via video conference or by telephone) to the exact procedure, the contents, as well as data-protection issues by a research assistant. Study participants kept their diaries for 4 weeks and could contact the researchers at any time. The duration of the diary study was chosen in order to minimise participant burden, on the one hand, and to obtain a representative picture of the daily life of persons with implants on the other hand. This included both during-the-week and weekend records, which covered the time span of 4 weeks. This approach aligns with existing research (see, e.g., Ref. [21]).

Participants submitted their records on a weekly basis and could keep handwritten or digital diaries. In the handwritten format, each participant received blank templates and pre-stamped envelopes to return the completed diaries each week. Participants who chose to keep a digital diary received the exact same template in *Microsoft Word* format and could complete their entries using a computer. The individual diary parts were reviewed by the researchers upon reception, followed by a weekly feedback conversation. The final conversation served to review the entire recording period in order to evaluate the method, similarly to the GDs. Additional data generated by these conversations were documented by the researchers and included in the analysis.

### 2.4. Analysis

Data from the GDs and the DS (incl. the corresponding notes taken by the researchers during the feedback discussions) were analysed based on Bohnsack’s documentary content analysis [23] and grounded theory [24], whereby the grounded theory was the superordinate research style. Since the grounded theory implies a non-linear and iterative research process, data collection and data analysis were conducted in a circular process; the development of the diary study was based on a preliminary analysis of the data from the GDs. Once complete, the data from the GDs and DS were then analysed in a process combining the documentary analysis and the grounded theory. The aim of this approach was not only to methodically triangulate the data, but also to provide an *in-width* and *in-depth* analysis [25]. Inductive thematic saturation and sufficient depth of understanding was achieved in the analytical process of the data from the GDs and the DS. This process was performed in the following three consecutive phases.

***Phase 1: Reconstruction of the thematic outline by means of formulating interpretation (documentary analysis) and memos (grounded theory)***

The recordings of the GDs were transcribed, and the diary entries were put into a standard digital format. The handwritten diaries were typed so that a homogeneous diary collection of all participants was created. The three researchers (C.H., M.L and S.S.) independently reviewed the material line by line and reconstructed the central thematic lines. Thereby, two different levels of statements were differentiated—descriptive (what was discussed, e.g., situations, experiences, diagnoses, etc and analytical (why does this matter, e.g., attitudes, values, beliefs, etc.) [23]—and memos were recorded. The three researchers compared and discussed their work and collaboratively selected the themes and text sections that were to be included in the next analytical step.

***Phase 2: Exploration of the collective orientation patterns (documentary analysis) and open coding (grounded theory)***

In the second phase, the selected text sections were further analysed by means of the documentary analysis using reflective interpretation and collective orientation patterns were elaborated. Furthermore, the memos (recorded in the first step) were analysed and related to the founded patterns so that an additive interpretation took place. This step was again independently performed by each of the three researchers. The systems of collective orientation patterns were then compared, discussed and merged, so that an integrative system was finally created for each type of implant. Parallel to the reflective and additive interpretation, data were coded by means of open coding using the principles of grounded theory [24], so that the orientation frameworks could be further substantiated by different specifications of the pattern. Coding was divided among the research team, with each coded transcript being cross-checked by a different researcher. Any conflicts were resolved in discussions among the three researchers.

***Phase 3: Type formation (documentary analysis) and abductive reasoning (grounded theory)***

In the third phase, the authors searched for thematic cross-connections between the collective orientation patterns from the three different implant fields; at the same time, the different cases along the orientation patterns within each implant field were also compared, informing the type formation (documentary analysis). In this stage, the theoretical memos were also included in the analysis. In order to relate the data and the type formation to theoretical reasoning (already noted in the memos), explanatory hypotheses [26] (which were heuristic in character) were formulated (grounded theory). Each hypothesis disclosed specific collective orientation patterns which emerged in the second phase of the analysis (e.g., training on technology use) and was then related to known concepts of health literacy and ethical values (e.g., technology handling as part of the functional health literacy and perceptions on technology as a factor for self-determination). The resulting hypotheses on health literacy in implant care were then cross-verified along the transcripts. This step allowed for elaborating on the different types of health-literate behaviour following the principles of the documentary analysis [23].

### 2.5. Participants

Twenty-eight participants took part in the GDs and thirteen in the DS (total *n* = 41). One participant with cochlear implant and one participant with a cardiovascular implant participated in both methods. In total, 15 participants with cochlear implant or parents of children wearing cochlear implants took part in three GDs (*n* = 2 pre-lingual, *n* = 9 post-lingual and *n* = 4 parents of children wearing cochlear implants); 8 individuals suffering from glaucoma took part in two GDs (*n* = 6 no implant and *n* = 2 with implant); 3 wearers of cardiovascular implants took part in one GD (*n* = 3); two further participants with cardiovascular implants could not actively participate in the GD due to technical difficulties or bad internet connection and were additionally interviewed via telephone (*n* = 2) along the same question script used during the GDs.

Regarding the DS, 7 participants wearing a cochlear implant or having a child with cochlear implant submitted their diary notes. Moreover, 4 individuals suffering from glaucoma and 2 participants with cardiovascular implants participated in the study. The sample characteristics are described in Table 4.

## 3. Main Findings

The major collective orientation patterns for all three clinical fields refer to (1) information and individual perceptions; (2) appraisal, dependence and responsibility; and (3) implant-related values. The further specifications of the patterns vary for each type of implant, according to the specifics of the implant, the therapy or the disease, as well as the two qualitative methods. The collective orientation patterns and their specifications can be found in Appendix C, Table A3.

### 3.1. Information and Perceptions Regarding the Implant, Technology and Disease

Data analysis revealed the perceptions of the implant as well as one’s own attitudes towards medical technology as collective orientation patterns, in the context of perceptions regarding the implant, damage prevention, control over one’s implant and health in everyday life, and finding and dealing with information, especially with regard to decision making. This not only refers to the knowledge and information about the implant, its handling and user experience, but also to the perception on the implant in relation to the body.

#### 3.1.1. Perceptions on the Implant as a Physical Object

In the case of **cochlear** implants, the implant was perceived as part of the body and wearing it evoked a sense of normalcy blending in with everyday life. Participants described their perceptions on the implant as follows:

*“[…] to the extent that you experience that the technology becomes part of you, that is also fascinating […].”*

(GD1CIA9)

*“You can simply take part in life in a normal way […], is like a pair of glasses, that you simply put on and then go through your everyday life in a normal way, with no restrictions, but having a technical device with you without really noticing it”.*

(GD2aCIB2)

A mother of a child wearing a cochlear implant described the CI as “a piece of jewellery” also demonstrating her positive attitude and presumably also the positive attitude of her child towards the implant device.

Similarly, to implant wearers with a CI, implant wearers with a **cardiovascular** implant described:

*“The implant/band is part of oneself, completely normal, it is like wearing glasses.”*

(DC2)

For implants that are physically less visible, such as **glaucoma** stents, the data demonstrate that the implant itself was hardly noticeable. A participant described this in the GD as follows:

*“I do not perceive the stent as a foreign body or in any other special way.”*

(DGl1)

Nevertheless, the following explanation of a GD participant demonstrates that, even if the implant itself is not perceived physically, it could be indirectly noticeable through certain accompanying symptoms:

*“I have to say that these XEN stents have created filtering blebs in the eyes, […] not formed intentionally, and they are also on the surface of the eye […], so that when you blink, the eyelid rubs over them, which is a mechanical irritation every time. That is what I experience as a direct consequence of these implants.”*

(GD2GlB1)

#### 3.1.2. Perceptions on Implant’s Functioning and Damage Prevention

Besides perceiving the implant as a physical object within the body, the perceptions on the implant’s functioning, combined with the relevant information and knowledge, play an important role in everyday life and well-being. In the case of **cardiovascular** implants, this became especially apparent with the implant and its technological functioning being perceived as one of two extremes: supporting and enabling versus thought-consuming and inhibiting. On the one hand, the perception of the implant as reliably functioning and failure-free can elicit a certain level of trust and feeling of security in one’s own physical body and establish a sense of normality. This was described by a participant as follows:

*“After the successful tape-laying my optimism returned and today I definitely have the feeling (wrongly?!), that at least I will not die of this one day. I have a feeling of invulnerability in this area, as stupid as that may sound! Despite all existing physical limitations, also because of my age. But it is a good feeling !!!”*

(DC2)

On the other hand, worrying about a possible implant failure can become very consuming in everyday life. The concern about possible damage to the implant due to stress was particularly relevant; in the same vein, the implant itself was also perceived as a source of stress or anxiety and therefore, in a way, also harmful for its own functioning:

*“I am afraid that this permanent stress (caused by the implant) will damage the implant. Actually, this thinking determines the day. This question comes up all the time.”*

(DC1)

Being aware of the implant and the perception of its functioning is a prerequisite for another aspect of implant care which was also mentioned in this quotation: damage prevention.

For persons with **glaucoma**, damage prevention mainly refers to preventing the progression of the disease in order to avoid blindness. In this regard, a possible measure is controlling the intraocular pressure either with an implant or with drop therapy, which may be necessary in addition to the implant. Participants regularly wrote about drop therapy in their diaries, describing the administration of eye drops as a kind of ritual (1) and depicting its integration in everyday life (2):

*“The evening ritual. Left Trisopt right Xalacom dropped.”*

(DGl4)

*“Eye drops are always in the bag and a reminder is set in the phone so I drip every three hours.”*

(DGl1)

Since intraocular pressure cannot be perceived physically, there is no direct way for implant wearers to know if the pressure is regulated effectively. In the context of damage prevention, this gave rise to feelings of uncertainty as well as the desire to gain more control over the measurement of intraocular pressure and thus over the disease. An increased interest in technical innovations was communicated during the discussion, paired with scepticism towards the state-of-the-art glaucoma treatments in medicine:

*“[…] aren’t there ways to measure intraocular pressure constantly over a longer period of time [...]? That would primarily be a question of a reliable and self-applicable technique. Are the researchers from the university perhaps better informed? I haven’t heard anything about it yet, but such technology would perhaps be also a way of guiding patients towards [...] being able to control themselves better with this data, instead of blindly relying on [...] data collected in one single point of time (during the medical check by the doctor).”*

(GD1GlB2)

Similar to the case of glaucoma implants, self-monitoring and self-knowledge were important assets among participants wearing **cardiovascular** implants when it comes to controlling disease progression and maintaining implant functioning:

*“I also measure myself, my coagulation value every week. I know exactly where there are risks, where there are no risks and what is just as important […].”*

(GD1CB2)

*“I’ve been keeping detailed records since I was discharged from rehab after the heart attack: weight, diuretic dose, blood pressure, exercise profile. So that I can recognise a connection in case of possible strong changes.”*

(DC2)

With regards to **cochlear** implants, damage prevention mainly refers to the prevention of material damage to implant parts such as the speech processor and batteries (not waterproof). The concept of damage, here, was more on a technological level related to external influences in everyday life:

*“X (*diarist’s child*) is missing her processor of the second CI for a third day in a row. We had quests on the weekend and there was a water battle […]. X got so wet, so that one of the battery cases got also a load of water. Since Friday evening, X is only unilaterally supported by the CI.”*

(DCI7)

#### 3.1.3. Information and Knowledge Related to the Implant and the Disease

With regards to cochlear implants, individual’s general interest in innovation and positive attitude towards technology may incite efforts to improve the quality of hearing with the implant, as well as the wish for a more precise technology adjustment. One participant (pre-lingually deafened) described this as follows:

*“I’m […] interested in innovations, I also look at something newer from time to time, but what would be even more interesting for me would be if you can make more […] progress in the [...], technical settings. That you can make even finer adjustments to the sound quality, [...] that would be even easier for us.”*

(GD2aCIB1)

The capability to avert implant failure, or ensure or optimise (in the case of CI) functioning presupposes sufficient information and knowledge about the implant, technology and disease and empowers patients to handle the implant and disease in everyday life.

Overall, information seems to be mainly obtained through internet research, exchanges in a private context (self-help groups, family, friends, random encounters, etc.) or consultations with health professionals. Participants with **glaucoma** refer to the internet as an important source of up-to-date information that was also considered reliable (1); participants also reported positive developments (especially in recent times) in finding adequate information online (2):

*“[…] I have informed myself mainly* via *the internet, the glaucoma forum was essential. You can find really good information there. Then also on the university pages. And I tried to read some of the publications.”*

(GD2GlB2)

*“[...] it has somehow become clear to me that medical knowledge changes significantly over the years and the assessments of it, so that it is good if you try to keep up to date as intensively as possible, and of course that is better today than it used to be via the internet [...].”*

(GD1GlB1)

A participant in the **cochlear** group discussion also referred to the internet as a source of information in interaction with healthcare professionals and described:

*“I’m on the internet a lot and find out about things on the internet or through other contacts, if I’ve picked up something new somewhere, I look it up more on the internet and when I’m stuck, I ask experts who might already know more about it and ask where I can look up something else.”*

(GD2aCIB1)

In light of the fact that, e.g., negative side effects of the implant are not always communicated transparently in the care system (see Section 3.2.), the exchange in the peer group was also considered important. One participant wearing a CI (post-lingually deafened) explained:

*“I find that increasingly important, I mean I know my family, [...] we have a lot of experience that we can exchange, but for example the neck tension, which I have only just learned here that it also affects others, I just don’t know that and from the XY [healthcare institution], [...] so far I have been rather dismissed that it doesn’t come from the CI. I think it would be nice if there was [...] a closed platform where you can exchange [...].”*

(GD1CIA6)

The notion was similar in the **glaucoma** group discussion; information from the peer group was also considered crucial in general, where the self-help groups were seen as a space for information exchange, networking and discussing (1) and were also perceived as empowering for participants in terms of decision-making processes (2):

*“[…] for me, the self-help group is clearly a tool to inform myself [...] the self-help groups are an organisation that already exists and where you can definitely network, where you can query, where you can discuss.”*

(GD1GlB4)

*“I (have) talked to many patients [...] who just had an operation [...]. Then I had the feeling: “Okay, I can assess a little bit what people experience” and then I also talked to the doctors-they also wanted to do a seepage cushion operation on me. [...] So 15, 16 years ago, there were at least half of the seepage cushion surgeries that didn’t work. And since it was absolutely necessary for me, I had the feeling: “Well, now I’m smart enough and I can assess it for myself” and then I said: “No, I don’t want to”, because my pressure values were really good. [...] I got information from my fellow patients and then I did a lot of research on the internet and through the self-help group […].”*

(GD1GlB3)

Similarly, the *Heart Foundation* was perceived as an important source of *information* for participants with **cardiovascular** implants. It offered an opportunity to exchange information and to gain technology- or disease-related knowledge through peers:

*“That’s why I went to the Heart Foundation, there’s a lot of information there [...]. Of course, I know that there are people who see the whole thing more casually, according to the motto the doctor has to make me healthy, but after my valve operation I had a rehab that was especially for people with heart valve diseases and you could see that most people had already dealt with it [...] that there are possibilities and I like to take the information from the rehab to avoid further damage, […], to keep myself fit and to get the best out of the situation.”*

(GDCB3)

Attitudes towards implant technology (see Section 3.2) interact with the medical and technical information on the implant and were considered essential for decision-making. As a result, participants felt the necessity to inform themselves as extensively as possible. A parent of a child with CI described the feeling of being left alone with the decision and explained:

*“[…] it was an incredibly difficult decision. I also obtained information where it was available, but as a parent you are relatively alone, and it’s a decision that you don’t make for yourself, but for your child, and there are also certain risks, and not just the health risks.”*

(GD2bCIB2)

The complexity of this information environment is also characterised by the high speed of technological progress in the **cochlear** implant area. This puts implant wearers in the position of informing their doctors on technical features and functioning. A participant wearing a CI said:

*“There are different implants, they are always developing and that is of course important, because I always feel as if I have to inform the doctors about what works and what doesn’t work or which direction it goes in or what it does to you.”*

(GD1CIA1)

In the context of a perceived lack of knowledge of doctors, however, a diarist with **glaucoma** reflected on uncertainty in the research context in one of the feedback interviews; in their opinion, receiving uncertain or lacking information results from low levels of existing knowledge about the disease and its causes in general, so that doctors cannot make any well-founded statements. A study author took field notes during the telephone call with participant DGl1 after the first week of diary keeping and made the following note:

*“Uncertainty among patients (and doctors) is caused by a lack of research into the causes of glaucoma. Since the causes are not known, it is difficult to assess the chances of success for the treatment as a whole. Control of symptoms works to some extent, but it is difficult to assess how and with what prospects a progression of the destruction of the optic nerve can be prevented. The patient sees the reason not so much in the lack of information by doctors, but in the fact that doctors themselves cannot make precise statements and recommendations because there is a lack of knowledge and research in this area.”*

(study author Sa.S)

Against this background, participants wished for a more holistic approach regarding glaucoma, as one participant in the GD stated:

*“[…] that you also perceive the eye as a component of the brain and the whole body, and that you have to make sure that it is also properly cared for. [...] That not only the purely mechanical treatment in the eye, but also this peripheral view should be expanded”*

(GD1GlB4)

In light of a perceived lack of knowledge about the disease, participants felt the desire to contribute to the research processes by providing information on their individual disease peculiarities. A participant in the GD explained:

*“What would really be close to my heart is to find more cooperation, with researchers on the subject of glaucoma, because there are so many glaucoma patients who have now also decided to get informed and to observe themselves with their peculiarities in relation to glaucoma, so I think we could really contribute a lot to this, because the doctors don’t have glaucoma themselves and we know a lot of what they don’t know.”*

(GD1GlB3)

In the group of **cardiovascular**-implant wearers, gathering information about surgery conditions and techniques was very important for participants, since it enabled them to assess their own needs and wishes concerning the therapy. The process of decision making in view of the prevailing medical assessment versus individual fears and uncertainties was described by one group discussion participant as follows:

*“When the heart valve doesn’t work as it used to, then you get weaker, [...] it’s a long process over many years and then my doctor said, “well, you know, you have to deal with it, in your case it can be done quite well today, the heart valve can be replaced quite well”. [...] then you get to know what that means, you are cut open from the neck to the navel, then the whole chest is opened up, [...] and (as I) was already afraid of this thing, [...]* (the information about this minimally invasive operation method) *came naturally just at the right time, then I enquired, they told me “you are too young”, “what does too young mean? “I said, “I’m 77 now”, “yes, we don’t actually do that until you’re over 80”, and so I looked into it and [...], the Heart Foundation offers all kinds of information, not just the material on the website or the brochures they have, you can also talk to cardiologists there, and so it was clear to me from the start that if it’s an option for me, I want to have it.”*

(GD1CB3)

Feeling well informed about the implant and the disease empowered patients and reinforced their efforts to seek, find and communicate health information. Evaluating such information adequately and applying the gained knowledge and experiences in the care context were considered essential for the successful management of the health condition. This process also raises issues of (critical) appraisal and one’s awareness of (in-)dependence and responsibility, which are presented in the next section.

### 3.2. Appraisal, Dependence and Responsibility

The comprehensive appraisal and application of gained knowledge and experiences are considered essential for coping within the healthcare setting. Participants repeatedly described their experiences of insufficient counselling, trust in patient–doctor communication and a lack of transparency regarding the differences in quality of care. In particular, taking an active patient role was described as an important skill.

#### 3.2.1. Appraisal of Information and Disease

A particularly important issue for participants with **glaucoma** was glaucoma care itself; it was perceived as illness-centred, determined by an isolated view on the eye leaving scarcely any room for a holistic approach. Such an illness-centred approach was perceived as unsatisfactory by the participants and evoked frustration towards the treatment environment. This resulted in a feeling of being solely individually responsible for one’s own health (1) and negative experiences within the doctor–patient relationship expressing a perceived lack of sensitivity and belittlement as follows (2):

*“You shouldn’t leave everything to the doctor [...] ... I can only say from my experience that I would already be dead if I had always followed the doctor’s advice, and you also have to listen to your own gut feeling about the story very carefully, because the doctors don’t know you that well. So, you have to deal with the subject yourself and not leave everything to the doctors.”*

(GD1GlB5)

*“Yes, well I noticed at our glaucoma meetings that many ophthalmologists simply dismiss it, do not answer questions and simply downplay the whole topic.”*

(GDGl1B5)

Participants in this group not only felt responsible for informing others about glaucoma but also for appraising relevant information in an opaque information environment with many different sources. In a diary feedback conversation, one participant (DGl1) with glaucoma also emphasised the struggle of critically appraising information and making up their own mind against many different opinions. Against this background, exchanging information within the peer group, or with friends, acquaintances and doctors present important means of appraising information.

Among participants with **cardiovascular** implants, the process of appraisal was weighted differently depending on their medical history: participants who had a sudden cardiac condition and concomitant emergency surgery were engaged in appraising their health in line with cardiac-disease prevention and healthy living only after the implantation (1); participants who were aware of their heart problems were also concerned about a healthy lifestyle but had had additional time to appraise recommendations and risks on surgery options in more detail and actively partook in their care provision (2):

*“I mean, for a year now I’ve been thinking about almost nothing but health and about [...] doing everything to live healthily in order to grow old and have a good quality of life, and health is something I can perhaps influence myself by trying to live healthily and yes, it’s actually about that every day.”*

(GD1CB1)

*“I wanted to keep my heart valve at all costs, I wanted it to be repaired, then after a long search, [...] I found a hospital [...] where the head doctor reconstructed the heart valve. He told me at the time that the chance was about 50%, but I did it anyway and it went wrong, [...] Well, after five and a half years I had to go back to the operating table and then I got the mechanical heart valve [...]. Since then, things have gone uphill, and then I decided to pass on my knowledge so that other people don’t have to search like that, and I applied to the Heart Foundation and started working there straight away, and the heart valve I have now, I can live with it, I can cope with it, I know what’s going on, [...].”*

(GD1CB2)

The last quotation also suggests that the patient’s role in the process of knowledge appraisal (active demanding versus passive receiving) may determine both the perceived quality of care and the patient–doctor relationship.

#### 3.2.2. The Role of the Active Patient

The participants in the **cardiovascular** GD described how they perceive their role in the process of knowledge appraisal and explained how they prepare themselves for medical consultations in order to be able to critically question a doctor’s advice and recommendation:

*“[...] you have to [...] educate yourself, you have to inform yourself, you always have to learn and that’s what I do every time, now I have another routine cardiology appointment on Monday and I’ve already written down some questions and you just have to and that’s what I’ve learned, that it makes sense if you present yourself as an educated, informed patient and not like an idiot who listens to everything they say, how great it all is. […].”*

(GD1CB1)

Similarly, in the **cochlear** GDs, the importance of assuming an active patient role–being proactive in care management, claiming certain services and taking responsibility in the context of provision of care—was accentuated. This was perceived as a prerequisite for imparting empowerment:

*“I lodged an objection and explained to them on two pages my facts as I see it [...] and that I don’t think it’s okay that the health insurance company thinks otherwise and that it wouldn’t be vital for us* (decision for or against financing the aqua-case), *and I explained my thoughts to them and got it accepted [...].”*

(GD2aCIB1)

The importance of individual assertiveness and communication skills regarding the equity in quality of care was also described by participants suffering from **glaucoma**. For effective dealing of the disease, it was considered essential to be able to claim adequate care, initiate diagnostics and treatment, as well as to obtain sufficient counselling:

*“[…] there are also people in our group who can’t articulate themselves so well verbally or who are rather quiet and reserved and they won’t get what others can enforce because they [who are more articulate] can deal better with the doctors.”*

(GD1GlB3)

*“[…] if you can’t open your mouth or you don’t know what question to ask the ophthalmologist, then you’re in trouble.”*

(GD1GlB6)

A related topic discussed among participants was the doctor’s reaction to such an active and assertive patient role. In the case of participants suffering from glaucoma, the varying quality of care, the low transparency with regard to the experience of the clinics with MIGS and a lack of empathy hampered the coordination of treatment. One participant explained:

*“*[doctors] *react in an offended way […] when you have already been somewhere else, and perhaps also in another clinic [...]. I have experienced several times that the doctors react very insulted: “Oh, you have already been somewhere else, so we won’t do anything more for you, because then you should go to where you have already been”.*

(GD1GlB1)

In the case of **cardiovascular** implants, the coordination of therapy and aftercare was considered as a decisive component for successfully dealing with the disease. One participant evaluated his experiences in retrospect:

*“Yes, there’s a world of difference between having it* (the implant)*, wanting it and getting it (laughs), it was a long process. I applied and then they told me, “[…], pay attention, so this is a relatively new procedure”, [...]. I got it, it went well and […], even if I were to fall over now, I’ve lived with it for two years [...] I’m glad that I have it and hope that it’s a [...] biological part in there, but I didn’t even ask whether it’s from pigs or cattle, I know that it’s been adapted with my blood and set up, so that’s how I got it.”*

(GD1CB3)

#### 3.2.3. Dependence on the Healthcare System and the Implants

Considering patient’s dependence on good care quality, experiences with insufficient consultation were perceived as frustrating. The need for assertiveness seemed to arise from a feeling of *dependence* on the healthcare system and the implants themselves. With regards to **cochlear** implants, the feeling of dependence directly relates to the production of manufacturers (functionality, technical state and range of functions of the respective implant-make) and indirectly to the access to alternative care services (after implantation). Participants of the cochlear GD described this as follows:

*“I got the first (implant) in 2012 and now the second in 2020, even though (my hearing) was actually already very bad, I waited so long because I always had [...] in the back of my mind that I was making myself dependent on the technology […].”*

(GD1CIA6)

*“We are not unhappy, but it is still the case that we would not have the chance to say that we are no longer happy, so we’ll just change. […] we are dependent on the implant manufacturer making the same technical progress as the others, so that we don’t always look enviously at the others and see what they have just developed, but that they [...] catch up.”*

(GD2bCIB3)

A similar feeling of dependence on technology and manufacturers, but particularly with a focus on participation in certain activities of everyday life, was described in a diary entry by a cochlear implant wearer:

*“Artone 3 Max headphones that connect to CI seem to be broken. I’ve noticed that I’m really lost without headphones; I hope that the problem can be solved quickly. I do a lot with headphones: I have a university seminar coming up I’m totally surprised by how much this thing with the headphones is bothering me, but technology is also pretty important at the moment & if one thing is missing that I urgently need, then the whole situation is pretty annoying.”*

(DCI4)

In the context of **glaucoma**, the issue of dependence manifested itself in drop therapy. Although applying drops was seen as an integral part of everyday life by most study participants, it was also referred to as burdensome with regards to the side effects of the medication or the necessity of continuous use. One participant of the GD described this dependence as follows:

*“I hope that there will be even better eye drops in the future, […] that you don’t have to apply so often and that this feeling of constantly having to think about it and this ‘the day is timed according to eye drops’ will simply decrease a little. […] ... You always have the feeling that you are never completely free of it, that there are only four or five hours in between the drops, […].”*

(GD2GlB1)

A dependence on the implant and accompanying medicinal care was also evident in patients with **cardiovascular** implants. Since there is an increased risk of mortality and physical limitations in the case of unsuccessful or non-treatment, “no treatment” or a decision against an implant is not a real alternative. This is especially the case when the implant is inserted due to an emergency. One participant described this feeling of dependence as follows:

*“[…] if I had stayed at home, I would be dead now [...] and in the meantime I’m learning more and more, I’m questioning my medication, because I also notice that some things just don’t really work [...], because I know that if I don’t take certain things, then I’ll feel bad at some point [...].”*

(GD1CB1)

#### 3.2.4. Attitudes, Coping and Responsibility

Acceptance was considered as a way of handling such feeling of dependence among both participants with cardiovascular and cochlear implants, although the mortality aspect was relevant only in the context of **cardiovascular** implants:

*“[...] I accept that I have heart disease and I’m grateful above all that I’m doing so well and accept this stent, so if I ... there I also thought about, that in principle I would probably accept everything that prolongs my life, or that helps me.”*

(GD1CB1)

In the case of **cochlear** implants not only acceptance but also a self-confident attitude towards the implant in interactions with others seemed to play a significant role as a way of coping:

*“Some people, if they don’t know me and my CIs very well, seem irritated and stop talking, even though I can still hear. If it happens that I have to change the battery, I say something like ‘I have to change my battery for a moment, I can still hear you with the other side, keep talking’.”*

(DCI5)

*“I usually also explain what I have on my head, because the devices stand out and people don’t dare ask questions. So, I explain it proactively, which always goes down well with the counterpart and also has a likeable effect.”*

(DCI3)

Another way of handling the feeling of dependence was gaining control over the knowledge on medical issues and participants’ own body. Participants with **glaucoma**, e.g., were well-versed in the technical language regarding the glaucoma disease and saw this as a part of self-responsible disease management. One participant self-monitored their vision to prepare and inform future medical appointments and to be aware of variations in their visual acuity. The results were meticulously (self-)analysed (comparing both eyes, controlling the vision during different activities and times of day) and described in the diary as follows:

*“Distance visual acuity left as good as after standing up (=very good), right noticeably less than after standing up. Visual acuity at medium distance (approx. 3 m) good on the left, surprisingly worse on the right, ditto even for short reading distance (50 cm), where the left visual acuity was good, the right at best sufficient. Reading on the computer clearly better on the left than on the right.”*

(DGl3)

In the case of **cardiovascular** implants, being well-informed in terms of individual *responsibility* was manifested regarding individual risk–opportunity assessments of one’s own physical capacity, life planning, healthy-living choices and experience-based acquisition of medical knowledge:

*“I used to do a lot of sports, […] but in that respect you do think about it, whether you go a bit further away for skiing or whatever, it doesn’t really have much of an effect on my normal life, it’s just that you’ve become more cautious when it comes to taking risks or going further away, which is what you used to do.”*

(GDCB3)

*“I have also given some cardiologists further training, that is, when I go on holiday to other countries, for example warmer countries, [...], different diet, that my coagulation value changes again, just from the temperature, I should know that, if I get diarrhea what do I do there, but if you measure yourself, you are always on the better side and then you can help yourself. [...].”*

(GDCB2)

Effective interaction within the implant care setting requires an adequate and well-balanced appraisal of information. Firstly, being well-informed (Section 3.1), secondly, being able to critically appraise and apply information and ultimately, to be assertive within the care environment are factors that lead to a certain degree of independence and support patients to act responsibly in their own care management (Section 3.2). All these aspects influence and are influenced by ethically relevant (individual) values. These interactions are described in the next section.

### 3.3. Implant-Related Values

The data of this study illuminate the role of some value-laden issues in implant care related to the impact of implants on self-determination, irreversibility of the treatment, perception of emotional and physical burden in everyday life, identity and vicarious decision making, equity, participation, and discrimination experiences. These were differently weighted and represented among the three different groups of participants according to the disease and type of implant.

#### 3.3.1. Self-Determination in the Context of Treatment Irreversibility and Perceptions of Good Life

In the case of **cochlear implants**, the fast pace of technological development paired with the irreversibility of the implantation impeded the process of decision making. Thereby, the execution of patient autonomy in the sense of self-determination can be strongly influenced by this circumstance. Participants described challenges in adequately assessing the consequences of living with an implant in general as well as in regard to a specific implant brand. A participant wearing a CI described this as follows:

*“[…] the brands of course have very different options, and when I got my first implant in 2012, I was [...] informed a bit about what options there are and what was recommended for me [...] and I also understood everything and the technology, but nevertheless it wasn’t clear to me at that moment what I was choosing […] and also not what the consequences were. [...] That’s fixed and because it’s in your head you can’t change it. You can’t just say ‘I’m going to go out and buy something new’, because that doesn’t work with the implant, but you have to live with the choice you’ve made, maybe by chance. Self-determination was not possible at that moment. Of course, it’s not possible to look into the future, you don’t know which manufacturer will be the forerunner at some point […], I know that too, but this self-determination is actually lost at some point, where no one can do anything about it, but I find that considerable and also very unjust in part.”*

(GD1CIA6)

This quotation also suggests some ethically relevant issues that challenge individuals in the process of *deciding for* and *living with* an implant: the feeling of injustice due to the perceived randomness of the technological up-to-datedness of implant delivery and probable inequity in implant care.

In the case of **glaucoma**, value-laden issues were mainly discussed with regards to subjective perceptions of a good life and the fear of blindness in the context of decision making on therapy and health management. The data show that dealing with the uncertainties around the glaucoma, especially in terms of its causes, influencing factors and prognosis, were perceived as limiting, both on an emotional and physical level. In particular, participants felt driven in their own care management by the impending loss of sight. Two participants suffering from glaucoma (without implant) described:

*“[…] I think the quality of life is limited [...] So you are afraid of losing your eyesight completely on the one hand and that can sometimes lead to you sleeping very badly over a certain period. And the other thing is that if you really can’t see well anymore, then you can’t do everything. [...] for example, photography [...], that’s also one of my hobbies, you can’t do it as well as with two functioning eyes. And in this respect, the quality of life is limited overall in a certain way.”*

(GD1GlB4)

*“I would have rather run to the ophthalmologist every day because I simply didn’t know what the pressure was like and it was very, very erratic and I couldn’t really live with it because it ... well, it pulled me down psychologically even more than I already was [...]. So, what do you do when the drops are no longer enough? Usually surgery, but if the surgery doesn’t work either-what do you do? So, you are faced with a very big dilemma […]”.*

(GD1GlB5)

Other participants did not perceive any impairments (in the sense of a subjectively lower quality of life) directly caused by the implant (1). Participants reported that they did not have any thoughts about the implant in everyday life, except when, for example, irregularities or uncertainties in the functioning were detected during a doctor’s check-up (2):

*“* *As the stent is not noticeable to me, it has no impact on my quality of life.”*

(DGl1)

*“When Dr. XY measured the eye pressure […], it was 19 in both eyes, which he thought was a little too high. It could be a small blockage in the implant. [...] If it was a blockage, it should be removed in about 6–9 months by a small operation [...] This situation moved me emotionally, because it would be an operation on my still better eye, and the fear that something could worsen my vision. It is strange that you have an implant in your eye and you think that’s it forever without any problems and then you find out that there might be a blockage without noticing anything like that.”*

(DGl2)

The findings regarding **cardiovascular** implants differed from the other two types of implants. This was primarily due to the fact that implant wearers were confronted with the possibility of dying due to their cardiovascular disease in the case of implant failure. The data predominantly provided insights in the role of anxiety, the psychological burden of participants’ encounter with their own mortality, trust in the technology and cohesion within the community. The fear of dying vis-à-vis the gratitude of being alive influences individual’s values and quality of life. One participant expressed his experience of gratitude as follows:

*“[…] I was totally grateful that I survived that, that I was so lucky, […], if I had stayed at home, I would be dead now and this gratitude then also subsided at some point […] at the end of the day, I have a high quality of life because I’m just grateful that I’m still here […].”*

(GD1CB1)

The data show that the medical history (emergency situation vs. already-known cardiac problems) determined which issues are relevant for the individual in the context of implant care. Furthermore, they emphasised psychological components that play a significant role in the everyday experience with the implant. Among the participants who were implanted as a result of a medical emergency, aspects of uncertainty (1), and disenchantment (2) were particularly significant, whereas participants with a known cardiac problem were able to reflect on and prepare for the implantation resulting in an increased sense of security and resilience (3):

*“[…] since the cardiac arrest, I was dead for about two minutes and my whole life revolves around safety, [...] and in principle I am constantly questioning whether I have to go to the emergency room again and [...] I don’t really feel safe.”*

(GD1CB1)

*“Since the operation, everything has been constantly going downhill. Only problems, […] that can’t be good.”*

(DC1)

*“I don’t have that* (fear)*, although of course you think about it, especially when you get arrhythmias again, but I’m not afraid in that sense. But I also suspect that it’s because I was able to prepare for it for a long time and wanted to have it and also got it [...] it still works, [...] I’m glad.”*

(GD1CB3)

#### 3.3.2. Identity and Participation

The data of this study show that concerns in terms of identity issues and participation also play a role in the process of decision making and handling life with an implant. In the case of **cochlear** implants, the general attitudes toward hearing impairment in society (especially when negative) may cause or reinforce tension and uncertainty. In the case of parents who need to make the decision on implantation for their child, some complemental factors directly or indirectly related to the implant and the hearing disability of the child come into play. These include, e.g., access to education and inclusion as well as language and identity in the context of the Deaf culture. A mother of a child wearing CI explained in one of the GDs:

*“[…], that we had to decide whether or not to go ahead with the implantation. That was also a big aspect for us, how does she deal with it, does she want it at all, because she was just at an age where she could not yet decide with us and we had to decide completely for our child and we have always said that was the most difficult decision of our lives, because everything else can be revised somewhere, but such an implantation sets somewhere a final point and the child must then organise its life with it. […].”*

(GD2bCIB2)

A mother of a child with CI described such emotional tensions as follows:

*“[…] as parents, we already had a stomach ache because we took this decision away from him, so I also documented it [...] in a letter [...] so that we could show him why we made this decision. [...] But of course we did it on the advice of the doctors, so that he would benefit as much as possible and later [...] be able to live a more self-determined life, because he would have better hearing. […].”*

(GD2bCIB3)

From a health-related perspective, belonging to a certain group of individuals who share similar experiences plays a significant role among implant wearers regarding their identity. Being part of a self-help group, e.g., meets the need for commitment and agency.

In the case of **cochlear** implants, such belonging is strongly connected to the Deaf culture or the ability to communicate in a way that allows participation. Especially among pre-lingually deafened individuals or parents of children who obtained an implant at an early age, such belonging is related to communication (communicating equally in both the hearing and non-hearing world):

*“[…] it is very important for her education that she learns sign language. At the same time, she also learns a little bit to deal with other children outside of the school environment in the normal sphere or normal life […].”*

(GD2bCIB2)

In the case of participants with cardiovascular implants, it became apparent that a heart disease and the accompanying confrontation with one’s own mortality increased participants’ need for exchange with other affected persons and promoted one’s own commitment. The participants with **cardiovascular** implants explained that shared experiences with cardiovascular diseases contributed to the development of a (partially life-changing) collective identity of those affected. It was perceived that this factor reinforces cohesion and self-conception within the group:

*“I’m also a member of the Heart Foundation and I’ve noticed that all the people who are somehow involved with heart problems are very special kind of persons who are incredibly helpful and simply loving […] ... I can call them all and they help each other and also the professor I’m in contact with there, they’re all really nice people and that’s why I say heart is something special.”*

(GD1CB1)

These quotations disclose one further significant ethical aspect of implant care—social participation and inclusion in different areas of the life of individuals with health impairments. One participant (post-lingually deafened) with a **cochlear** implant also related to experiencing tensions in social interactions due to their impairment and wrote in their diary:

*“Either I counter, or I withdraw. Already at home I was not “welcome” with my hearing impairment and rather an outsider. Others only talk to my wife, even about me. Even when I’m standing next to her. According to the motto: He can’t hear anything anyway.”*

(DCI1)

Despite negative and discriminating experiences in social interactions, all study participants wearing cochlear implants reported that being able to hear by means of the implant strengthened their ability to stand up for themselves and live in a self-determined manner, reinforced their self-confidence, and increased their autonomy. One participant described their experiences as follows:

*“Yes, you can stand up for yourself again [...] and that makes the whole thing fairer with the implant. That was always the problem beforehand, especially in my professional life. I was always a bit ignored or I couldn’t stand up for myself because I just didn’t understand that, or [...] then the situation was already over and this way you can [...] defend yourself better […].”*

(GD1CIA1)

Likewise, the **cardiovascular** implant enabled individuals to participate and regain activities. One participant also described feeling like a part of the community again because of the improved health:

*“After the rainstorm of the night, the traces of the flooded underground car park were removed together with the house owners. This took several hours and required some persistent physical effort. Without an implant, I would have not been any help to the community, I realised. I did not have air or endurance problems.”*

(DC2)

Comparing the data among the three different implant groups shows that ethically relevant values may differ, depending on the specifics of the implant technology or on the disease. Nevertheless, it became clear that ethically relevant dimensions in implant care and in life with an implant play a major role in both individual’s experiences in everyday life and the shaping of their lifeworld in all three implant groups.

### 3.4. Synthesis of the Study Results for Each Implant Type

We mainly gained insights into the individual perceptions on technology and health, sources and appraisal of information, the factors influencing dependence and responsibility, and individual values. These aspects influence decision-making processes, health behaviour, but also self-perception in the sense of one’s own identity. In the following table (Table 5), some key findings for each implant field are summarised.

## 4. Discussion

This study shows that individuals suffering chronic conditions where implants pose a therapeutic option see HL beyond the context of medical correctness concerning health or implant information and decision making. In addition, HL is related to the subjective appraisal of knowledge and information around the implant. These aspects are connected to the individual values regarding life with an implant and likewise affect them. Therefore, discussing HL in the context of implant care requires the illumination of the HL concept in its different facets regarding (1) dealing with information on the technology and the disease, (2) appraisal, dependence and responsibility and (3) ethically relevant values in the context of implant care. In the following, the main findings will be discussed along with the concepts presented in the introduction: functional and technology-oriented HL; communicative and navigation HL; critical HL. These concepts are discussed both on individual and an organisational level. Furthermore, these findings will be contextualised and compared with existing research in this area, despite the paucity of data in the research landscape on this subject.

### 4.1. Technology and Health–Individual Perceptions, Attitudes and Information Needs

According to our findings, in implant care, functional HL [10,27] needs to be extended and replenished with technological understanding and the dealing with technical information. Here, we mainly relate to individual HL. Nevertheless, it is also the responsibility of the healthcare system to support individuals in their efforts to enhance their functional and technology-oriented health competences. With regard to the dynamics of technological progress as well as the opportunities and risks of innovative technologies, there is a need for a pronounced tolerance of ambiguity. Furthermore, strategies for dealing with rapidly changing or insufficient evidence or information must be developed. Tolerance of ambiguity, as described by Norton [28], is understood as the ability to handle “information marked by vague, incomplete, fragmented, multiple, probable, unstructured, uncertain, inconsistent, contrary, contradictory, or unclear meanings” and to not automatically perceive it as a source of psychological discomfort or threat. Hence, patients must also be able to recognise the dynamics of technological progress and technological developments and consider the associated uncertainties (e.g., low level of evidence due to innovation). Patients should be enabled to understand and apply implants’ technological functionalities and corresponding background information to act in a self-determined and participatory manner.

Regarding **cochlear** implants, it is crucial that patients (or parents) comprehend the implant’s technology, functionality and its impact on daily life prior to implantation, to be able to appropriately assess risks and benefits, further treatment options and make an informed decision. A study by Wheeler et al. (2007) showed that such deeper understanding of technology was lacking, even though individuals could successfully manage the CI in everyday life [29]. Our study shows that, in the everyday use of cochlear implants, basic understanding of technology and functional range (incl. accessories) and awareness of one’s own responsibilities are essential for the successful use and protection of the implant in everyday live. Furthermore, patients *and* caregivers need to be aware of their own attitudes toward technology: especially in the light that one’s own attitude towards integrating technological devices in the body can shape both the process of decision making and everyday experiences with the implant. The ambiguity of technical or risk information in the context of implant care may cause psychological stress among implant wearers and challenge their functional HL. There is some evidence, for example, that young individuals wearing **cochlear** implants with a low tolerance of ambiguity worry more about technological hazards [30]. Other studies have also addressed technology failure and damage prevention [29,31,32,33,34].

In the case of **glaucoma** treatment (e.g., drops and/or implant), patients are challenged to understand the consequences for everyday life associated with the respective treatment option (e.g., regular administration of eye drops and check-up appointments). This is not only relevant to decision making but also for implant care. E.g., understanding the consequences of not taking the drops can motivate and facilitate adhering to daily glaucoma medication [35]. In this respect, other studies revealed that eye drops were a factor that reinforced the decision for implantation [12]. Compared to cochlear implants, the needed information is less technology-dominated, but decision making still requires awareness of the individual attitude towards eye stents and understanding of their function and surgery-related specifics. Since technical information often requires the use of complex language and specific terms, insufficient individual HL may be a “by-product” of differences in the levels of knowledge and spoken language [18] between patients and their doctors. Therefore, such insufficiencies do not necessarily reflect information deficits of implant wearers. Stress may be caused by regional–urban differences in the quality of care, insufficient knowledge about the cause of the disease and thereby correspondingly uncertain prospects for treatment success. This was also reported by another study that stated that glaucoma as a disease brings some degree of uncertainty due to its “unknown nature and symptoms associated” [12]. This is additionally aggravated by missing transparency regarding clinics’ implantation experience. Handling the ambiguity of such an information situation is part of individual HL and may support individuals in coping with stress.

In the context of the successful long-term disease management of **cardiovascular** diseases, it is essential for patients to develop strategies for dealing with stress (such as self-monitoring), i.e., to recognise their own needs and act accordingly. In addition, information about both the disease and the implant helps patients to manage their disease adequately. Furthermore, the skill of finding well-founded and sufficient information may increase individuals’ confidence and sense of security. Other studies recommended considering quality-of-life aspects and patient-reported outcomes when evaluating a patient for a certain type of procedure (e.g., SAVR versus TAVI) [13,36]. On an organisational level, it is important that health professionals create realistic expectations for patients, since the patients’ health and life might still be impacted by existing comorbidities [13,36]. In the case of heart valve surgery, studies show that there is not only the question regarding the type of procedure, but also the choice between biological and mechanical valve; too little knowledge and ability to assess information were seen as causes for difficulties in weighing up a decision [37,38]. Additionally, the peer-group exchange of information and individual experiences concerning, e.g., comorbidities or therapy side-effects, is perceived as helpful (which is in contrast to the findings of Schmied et al. (2015), that social support or peers were sources to which little recourse was made) [39]. Its promotion may indirectly contribute to the development of individual HL, especially in the context of existing comorbidities (since there is evidence that comorbidities among patients with implantable cardioverter defibrillators and pacemakers are associated with inadequate HL [40]).

Health prevention and promotion play a central role in patients’ HL. Therefore, knowledge about and the willingness and ability to implement health-promoting measures in everyday life form a fruitful ground for individual HL, but also require a certain degree of autonomy and commitment. In order to be able to meet patients’ pronounced need for exchange with peers, the healthcare system needs to inform about available possibilities, also outside the care setting. Decentralised platforms with curated information and possibilities of a direct exchange with professionals or self-help groups can promote needed skills for successfully handling and contextualising health and implant information.

### 4.2. Appraisal, Dependence and Responsibility–Building the Bridge to HL Competences

Our results show that patients can positively influence their implant care by assuming a demanding, informed and, above all, active patient role and exhibiting assertiveness skills. Such an active patient role may be associated with high individual HL, as a counterpart to the summary of evidence provided by Watson [3] on patients with low HL (asking few questions during medical consultations, lower adherence to medical advice, experiencing worse overall health outcomes). The study participants described that exactly those who act “more passively” and “less assertively” in the care delivery process have many more difficulties in obtaining adequate implant care. For example, appropriately assessing one’s own level of medical knowledge allows patients to build trust in those treating them and to meaningfully combine the provided information with their subjective risk assessment. Especially in implant care, medical knowledge and information can be ambiguous. Additionally, successful implant care and disease management require patients to (autonomously) deal with the disease and adjust their care management accordingly. This is driven by a pronounced degree of proactive behaviour, including the willingness and ability to obtain information and to co-shape the diagnosis and treatment processes in implant care.

Our results reveal two main dimensions of HL, which are considered to be essential prerequisites for implant wearers’ confidence in handling their health condition. On the one hand (1) communicative competences–as skills to participate in everyday life and to extract information and meaning making from different forms of communication and to apply this meaning to varying situations [9]. In addition, on the other hand, (2) navigation HL, described by Griese et al. (2020) as the ability to handle information in a way that enables navigating through the healthcare system on an individual level and in dependence of its complexity on an organisational level [10]. Both facets are related to the responsivity of the healthcare system to individuals’ information and negotiation needs (i.e., the nature of the doctor–patient communication and the officially distributed information, e.g., leaflets or presentations). Moreover, they are related to implant wearers’ needs regarding their efforts in navigating through the healthcare system and balancing among many different health services (i.e., coordination of the information flow between control examinations, additional therapies and hospital visits, as well as planning efforts and information needs). In the three different types of implants introduced here, the facets of communicative and navigation HL are emphasised differently on an organisational level.

From the perspective of **cochlear**-implant wearers, the ability of healthcare providers to enable participation and deliver responses to the needs of individuals related to their subjective experiences. This can incorporate, e.g., offering communication training or advice on how to deal with the new sense of hearing. In line with existing research [41,42,43], implant wearers had to adjust to the device and the “new” hearing experience with the implant, which required great effort. Furthermore, for successfully navigating through the healthcare system, implant wearers need to be able to understand and evaluate the importance of certain technical choices (e.g., the brand of the implant), make informed decisions on surgery or to decide among different health services and providers [32,44,45,46,47]. As outlined in the previous (Section 4.1) cochlear-implant wearers need information on technical and acoustics-related information. Such specific and often complex information require the system’s responsivity, offering training and advice for the ways of successfully navigating between medicine and technology. A study by Sach and Whynes (2005) reported that prospective implant wearers felt that they were merely handed off to the implant centre by their physician, which was perceived as stressful because it triggered uncertainty about implantation [48]. This aspect also correlates with technology-related HL (see Section 4.1). Another important aspect of organisational HL from the perspective of cochlear-implant wearers is the provision of spaces for the exchange of experiences and information with others who are faced with similar challenges, problems and questions, both regarding decision making [33,49] and the period after surgery [33]. A health-literate action on an organisational level is characterised, e.g., by providing sufficient information [44,50] that patients need for acquiring medical knowledge. This enables an informed interaction with the healthcare system [33,51]—being able to articulate one’s needs in a proper manner helps patients to make informed decisions on the variety of care options.

Since there is limited evidence on the success rate of the therapy with a **glaucoma** stent, the healthcare system needs to provide patients with specialists with high communicative HL who can assist patients in the process of risk assessment and decision making. Implant wearers emphasise their need to handle the glaucoma stent and the disease applying a holistic approach (glaucoma as a systemic disease) and wish to find this approach mirrored not only when searching for information themselves (see Section 4.1), but also by the healthcare system and the provided services. In line with these implications, studies indicated that the patient–doctor relationship plays a significant role where, especially, trust in one’s own healthcare provider and the perception of a shared decision-making process are influential factors [12,52]. The systemic character of the disease challenges individuals’ abilities to coordinate their diagnostic process and therapy and navigate through the healthcare system. Organisational HL should support individuals in their navigation efforts and offer paths for handling risk information based on scarce evidence.

With respect to passive **cardiovascular** implants, organisational HL is characterised by the transparent and comprehensible presentation of information, as well as low access barriers of care offers to enable patients to successfully coordinate their treatment. Since, in the case of heart diseases, patients are directly confronted with concerns regarding their own mortality and survival, organisational HL should be characterised by the responsivity of the health organisation to the individual and often vulnerable situation of the patient. For example, a study by Astin et al. (2017) found that, especially, the consultation between doctor and patient enforced the potential consequence of dying [13]. Moreover, concerns related to dependence on the functioning of the implant, psychological stress, risk assessments and mortality should be accounted for. Organisational HL should also include the provision of resources (information, time, offer of discussion) by professionals with high communicative HL to enable patients to deal successfully and make informed decisions in such an emotionally tensed field. This is highlighted by a case study of a patient, who reported that he felt overwhelmed and wanted to be “relieved of this decision”. Furthermore, he depicted the decision as a method to free himself from an adverse mental state regardless of the actual medical urge [53].

Organisational HL needs to enable empowerment and support patients in the process of navigation through the healthcare system (e.g., by providing relevant information, offering opportunities for discussion and enabling access to courses). Health care providers need to be aware of the individual situation of the (prospective) implant wearer, taking into account the (prospective) implant wearer’s dependence on the implant and the healthcare system, e.g., in counselling. This dependence is strongly related to the process of critically appraising information and individual responsibility. Therefore, the healthcare system needs to provide patients with sufficient space for negotiating individual responsibility and dependence regarding the provision of adequate patient care and thus promoting HL.

### 4.3. Implant-Related Values as Part of HL

The main findings of this study showed that knowledge about and the reflection on the implant may be a source of a subjective feeling of security (or insecurity) and self-confidence, strengthening the ability to act in a self-determined way in the care context. The lack thereof may have a negative effect on individuals’ care management and, thus, on the successful handling of the implant. In the process of care-related decision making in the long term, it is essential for both implant wearers and caregivers to be aware of their own moral values and convictions (e.g., in terms of technology and health, identity, disability and ability, and participation, etc.). It can be challenging for patients to reconcile the decision for (or against) an implant with their own moral values, life planning and self-perception. Uncertainties arising in this setting can become a heavy burden for some individuals.

In connection with proxy decisions for or against an implant, as is the case for parents’ deciding on a **cochlear** implant for their child, it is important to reflect on the ethical dimension of the long-term effects of the implants on the child’s everyday life and identity. Furthermore, the wish to provide the child (or oneself) with the best possible treatment as a precondition for a successful life is often accompanied by various other decisions regarding social participation, education, acceptance and inclusion, which need to be constantly (re)appraised. These aspects are also shown in other articles where parental decision making was perceived as emotionally burdensome [31,47,54,55]. Ultimately, the decision for an implant was considered as beneficial for the child [31,32,47]. Hearing with the support of the implant reinforces a sense of autonomy and implant wearers feel empowered. Through this, patients are enabled to act in a more self-determined manner (e.g., proactively claiming on health services), which is significant from an ethical point of view. This is also reflected in a study in which implants were used frequently by a younger generation, which was attributed to the fact that they are perceived as valuable for them [29].

In the case of **glaucoma**, HL enables individuals to act autonomously in the field of healthcare in accordance with their individual values and lifeworlds, e.g., while coordinating health services according to their individual feeling of security or proactively claiming for an early treatment. The scarce evidence regarding the therapy of glaucoma and the knowledge gaps in relation to the origin and background of the disease manifest in an emotional burden but also restrict the patients in their subjective quality of life. This is intensified by the possibility of concomitant drug therapies as a temporary alternative to the implant, which can hamper the patient’s decision-making process. This circumstance can present itself as a dilemma in the decision-making process. The handling of the disease requires a high degree of organisational HL (in terms of the responsibility in providing transparent und sufficient information) and individual critical HL (in terms of risk assessment and decision making) reflecting on ethical values. These findings are in line with Ontario Health (2019), which showed that individual values and experiences shaped the decision-making process, i.e., individuals’ perceptions of living as a blind person [12].

In the field of **cardiovascular** implants, HL is related to subjective quality of life and involves an informed and evidence-guided risk–opportunity assessment in accordance with individual and societal values and life planning. The trade off between lifelong drug therapy, open-heart surgery or implant longevity requires value-oriented thinking. In this context, implant wearers benefit from a strengthened HL in terms of tolerance of ambiguity, adaptability and acceptance. In regard to this, it was recommended that patients must be informed to be able to weigh risk and benefits adequately and to understand all short-term and long-term consequences [37,56]. Hereby, especially, the emotional needs and circumstances of older people in particular should be considered [57]. Individual HL enables patients to adapt to their new life situation with the implant not only on a physical level, but can also help them to understand and embrace their new identity. Organisational HL interventions should therefore offer spaces for considering the individual situation of prospective implant wearers during consultancy and decision making. In contrast to this aspect, other findings suggested that participants did not want to be part of the direct decision but rather, in general, be emotionally supported by friends [37,58]. Then again, some studies showed that health professionals were valued in the decision-making process as “co-deciders” who take part in the decision or even take it from the patient [39,53,59]. Astin et al. (2017) recommend to explore patients’ beliefs and preferences concerning quality and quantity of life in consultations [13]. This might enable patients to become aware of their values and create ideas of a good life, developing individual HL in this regard.

Against this background, given these diverse needs and preferences, value-oriented HL plays an essential role in informed decision-making, offering space for addressing the impact of the implant on an individual’s identity and lifeworld, including the notion of mortality or issues of social participation. HL can empower implant wearers not only in terms of self-determination but also in their educational role—in sharing their experience with and knowledge about the disease and the implant with individuals who face similar challenges. Such felt responsibility of the individual contains an ethical dimension, which should be embraced and framed within the context of care and for which a framework must be set. Value-oriented HL should increase awareness, especially with regard to the major impacts of implants on identity, quality of life and life planning. From a moral perspective, health organisations’ responsibilities lie in “examining and modifying their own activities, assumptions, and environments to remove HL–related barriers that hinder access to information, navigation of services, and decision making” [60], while constantly negotiating the system’s underlying values on implants, health and disease against the values of their patients.

## 5. Practice Implications

The discussion of the findings of the current article provides some starting points for the development of individual and organisational health literacy in the context of implant care from both ethical and patient-oriented perspectives. In the following, the communicated needs of the study participants and the theoretical conclusions of their analysis are boiled down to a list of recommendations for future research and practice (see Table 6).

On a political level, the insights from this study, including the ethical aspects of implant care, suggest the promotion of research and interventions on HL development regarding implants, to strengthen integrated healthcare from physicians and implant centres and, not least, to include the education of the communications skills of healthcare professionals in several stages of their professional development. Furthermore, ethical aspects should inform the technical development of innovative implantable technology.

## 6. Strengths and Limitations

A methodological strength of the study is that research gaps were narrowed in two respects: on the one hand, the development of a qualitative method, here the GD, in the online setting and, on the other hand, the triangulation approach of grounded theory and the documentary method in the analytical evaluation of the study. A method that is traditionally conducted face to face (GD) was successfully adapted and could be additionally evaluated. The evaluations were mostly positive. Since there is still little empirical evidence in the literature concerning online GD, this study contributed not only on content but also on a methodological level to existing research on HL and qualitative approaches [14]. Furthermore, the methodological triangulation by GD and DS was a strength in itself and condensed the collected data and findings.

Due to the pandemic situation during the period of study planning and conduction, the recruiting of study participants was aggravated. In order to increase the number of participants and include those who had technical difficulties and could not attend the discussion via *GoToMeeting*, the GD with cardiovascular-implant wearers had to be supplemented by two interviews, which led to a certain level of inconsistency in the methodical evaluation (even though efforts were made to minimise the discrepancy as far as possible). Moreover, due to recruiting difficulties, two participants (one cochlear implant wearer and one having a cardiovascular implant) participated in both the GD and the DS; in addition, in one case, the inclusion criteria for glaucoma participants were not as strictly adhered to (one participant had a normal-pressure glaucoma instead of open-angle glaucoma).

Concerning the study sample, few limitations need to be outlined. The sample size of the GD study was smaller than intended by the researchers due to the following two reasons: (1) wearers of glaucoma and cardiovascular implants were very hard to reach and in spite of the various intensive attempts of the researchers to reach potential participants, there were few individuals interested in taking part in the study and (2) the pandemic situation may have influenced individual’s willingness to take part in studies in general. One participant from the already very limited pool of participants with a cardiovascular implant appeared psychologically highly stressed, which may be an individual case and not necessarily representative of this patient group. Due to the significantly smaller pool of interested participants in the field of glaucoma and cardiovascular implants, the sample was not as diverse as in the case of cochlear-implant wearers (or parents of children with cochlear implants). For example, most participants with cardiovascular implants were male, which could be also due to the fact that cardiovascular diseases are still widely seen as male-typical diseases. As a result, such diseases are misrecognised in women or are discovered only at a late stage; the relative proportion of women who die from cardiovascular diseases is higher than of men [62,63]. It was also noticeable that all parents of children wearing cochlear implants who participated in the study were female, which can be seen as bias due to a gender imbalance in this sub-group. Furthermore, as the approach was based on open recruitment calls by project partners, and other institutions such as self-help groups, a bias could have risen from the fact that, mainly, people participated in the studies who are interested in their disease and concerned with their body and health.

A strength of the study is that there was no drop-out in either the GD or the DS. We attribute this to careful methodological preparation and close contact with the participants during the study period. The manageable number of participants in both parts of the study enabled the research team to continuously provide personal assistance to a high degree.

## 7. Conclusions

Given the innovation character of implant care in the context of chronic conditions, this study shows the role of implantable technology as a challenging factor for both individual and organisational HL. Individuals need to handle health- and technology-related information, staying up to date with the high speed of implant developments. Affected individuals need to find their way around the healthcare system, assess risks and act in a self-determined manner in the context of implant care. Furthermore, patients search for ways of integrating the implant in their everyday life, building on an emerging implant or disease-related identity and need to be supported in their efforts to reconcile their feeling of dependence with individual responsibility through critical and value-oriented appraisal of medical and technical information.

Such a complex interplay of competences, experiences and needs related to life with an implant requires from health providers to create efficient frameworks for orientation in a field dominated by ambiguous information and fast-changing evidence. From an ethical point of view, it is not enough to make implant care comprehensible, consulting implant wearers in a purely medical way. It must be acknowledged that an implantation may infiltrate various spheres of an individual’s body and lifeworld. Such *infiltration* touches on ethically relevant dimensions and values that need to be considered in the care context, increasing ethical awareness in the fields of HL development and in health-care practice. Finding place for reflection on the individual values of (prospective) implant wearers and the underlying convictions in implant care constitutes an essential task for organisational HL development.

Our study results demonstrated the interconnectedness between the acquisition and appraisal of technology-related information and knowledge about one’s own disease, the interactions within implant care between system and individual, the acceptance and adaptation of the implant in relation to one’s own body perception, on one hand, and ethically relevant dimensions such as dependency, responsibility and self-determination that accompany these aspects, on the other hand. Moreover, they emphasise the importance of individual and organisational HL, which must be, as a concept, sensibilised to ethically relevant dimensions in implant care.

For the development in individual and organisational HL, this demands a participative approach, and more attention to the technological layer of information and its role in supporting empowerment. Furthermore, if perceived as a hazard, the technology of the implant may impact HL and decision making. Interventions on HL should therefore provide advice and training on how to handle stress caused by too much, too complex or too ambiguous information. The continuous trade off between dependence (on the implant in everyday life and the health system) and responsibility (in terms of empowerment, decision making and critical appraisal) seems to be a core element of individual and organisational HL among implant wearers. Since values on health and disease play a central role in implant care, it is essential to pay closer attention to the ethical aspects of implant care and contribute to the promotion of a value-oriented HL. Since promoting HL may enable individuals to realise fundamental ethical values, HL development is an ethic responsibility itself.

## Figures and Tables

**Table 1:** Eligibility criteria for group discussions and diary study.

	Inclusion	Exclusion
Cochlear	Group 1: Post-lingual deafness and implantation; middle age Group 2a: Pre-lingual deafness and implantation in childhood Group 2b: Parents of participants from group 2a Minimum time after CI ^A^ implantation: 12 months Minimum age: 18 years	Age < 18 years
Glaucoma	Group 1: Drug therapy (drops) only Group 2: Micro-stent ± drug therapy (drops) Adults (≥50 years) with open-angle glaucoma ^B^ Minimum time after implantation: 6 months Minimum time of diagnosis and start of drug therapy: 12 months Visual acuity in the better eye ≥ 30%.	Age < 50 years
Cardiovascular	Cardiovascular implants Minimum time after implantation: 6 months Minimum age: 18 years	Age < 18 years
General in- and exclusion	Written informed consent of the patients Language skills: German language skills that allow participation in the study	Cognitive or physical limitations that do not allow study participation

^A^ CI = cochlear implant. ^B^ Due to recruitment difficulties of implant wearers with glaucoma, inclusion criteria were adjusted to normal pressure glaucoma and a minimum age of 18 years. The adjustment of the criteria applies to 2 individuals from the DS.

**Table 2:** Group discussions.

Implant	Group Discussion	*n* = 26 ^1^	Date of Realisation	Participants	Length
Cochlear	GD1CI	*n* = 9	8 December 2020	Post-lingually deafened	2 h
	GD2aCI	*n* = 2	8 December 2020	Pre-lingually deafened	2 h
	GD2bCI	*n* = 4	9 December 2020	Parent of a child with CI	2 h
Glaucoma	GD1Gl	*n* = 6	22 March 2021	Glaucoma	2 h
	GD2Gl	*n* = 2	24 March 2021	Glaucoma with stent surgery	2 h
Cardiovascular	GD1C	*n* = 3	29 April 2021	Cardiovascular implants (passive)	2 h
	Two individual interviews (Interview I2aC, Interview I2bC)		7 May 2021	The interviews were conducted with two persons who had technical difficulties and therefore could not participate in the group discussion.	1 h each

^1^ Plus two interviews.

**Table 3:** Diaries.

Participants	Time Period ^1^	Indication
**DCI1**	21 June–18 July	Post-lingually deafened
**DCI2**	21 June–18 July	Post-lingually deafened
**DCI3**	21 June–18 July	Pre-lingually deafened
**DCI4**	21 June–18 July	Pre-lingually deafened
**DCI5**	21 June–18 July	Pre-lingually deafened
**DCI6**	21 June–18 July	Parent of a child with CI
**DCI7**	21 June–18 July	Parent of a child with CI
**DGl1**	26 June–22 August	Glaucoma with stent surgery
**DGl2**	2 August–29 August	Glaucoma with stent surgery
**DGl3**	9 August–5 September	Glaucoma with stent surgery
**DGl4**	2 August–29 August	Glaucoma
**DC1 ^2^**	5 July–1 August	Cardiovascular implant (passive)
**DC2**	12 July–8 August	Cardiovascular implant (passive)

^1^ Data collection took place between June and September in 2021. ^2^ Participant DC1 also participated in an individual interview which was analysed along with the GD.

**Table 4:** Sample characteristics.

		Cochlear ^1^		Glaucoma		Cardiovascular ^1^	
		GD	DS	GD	DS	GD ^3^	DS
Total		15	7	8	4	5	2
Gender	Female	11	5	5	2	4	1
	Male	4	2	3	2	1	1
Age	18–30	2	4	-	-	-	-
	31–40	3	1	-	-	-	-
	41–50	4	1	-	1	-	-
	51–60	2	-	2	-	2	1
	61–70	3	1	4	2	-	-
	≥71	1	-	2	1	3	1
Living conditions	Alone	-	-	2	2	2	1
	With partner	6	4	6	2	3	1
	With relative	3	1	-	-	-	-
	With partner and relatives	6	2	-	-	-	-
Education	Abitur (graduated high school)	10	6	6	4	1	-
	Advanced technical college certificate	2	-	1	-	2	1
	Intermediate school diploma	2	1	1	-	1	1
	Secondary school diploma	1	-	-	-	1	-
Implant status	Implant wearer	11	5	6	4	5	2
	No implant ^2^	4	2	2	-	-	-
Marital Status	Single	3	4	1	1	1	1
	Married	10	3	4	2	3	-
	Widowed	-	-	-	1	1	-
	Divorced	-	-	1	-	-	-
	In separation	1	-	-	-	-	1
	n.s.	1	-	2	-	-	-
Cultural background	German	15	5	7	3	5	1
	Bi-cultural	-	2	1	1	-	-
Native language	German	14	6	7	4	5	2
	Other	-	1	1	-	-	-
	n.s.	1	-	-	-	-	-
Religion	Non-denominational	7	2	5	1	2	2
	Denomination	8	5	2	3	3	-
	n.s.	-	-	1	-	-	-
Occupation (multiple answers possible)	Healthcare	3	3	1	-	1	-
	Social services	2	-	-	-	-	1
	Science	-	-	3	-	1	-
	Economics	2	2	1	1	1	1
	Administration	3	-	-	-	-	-
	Commerce	-	-	1	-	-	-
	Industry	1	-	-	2	1	1
	IT	3	1	-	1	-	-
	Craft	-	-	-	1	-	-
	Art/Culture/Design	-	1	1	-	-	-
	Service	-	1	-	-	-	-
	other	1	1	1	1	1	-
	n.s.	-	-	-	1	-	-
Employment status	Employed full-time	4	2	2	-	-	-
	Employed part-time	4	2	1	-	-	-
	In education/study	2	2	-	-	-	-
	Retired	5	1	5	3	4	1
	Job-seeking	-	-	-	1	1	1

^1^ One participant in the group of cochlear and one participant in the cardiovascular implants participated in both methods. ^2^ In the case of cochlear implants, this accounts for parents of children with CI and in the field of glaucoma, this accounts for glaucoma patients without implants. ^3^ Incl. two interviews. n.s.—not specified, GD—group discussion, DS—diary study.

**Table 5:** Overview of the collective orientation patterns for each implant type.

	Selected Key Findings	Summary
**Cochlear**	Rapid development of technology Dependence on technological functioning (vicarious) Decision-making process Identity and participation	In the GDs, the decision-making process regarding the implant, keeping up with the ongoing technical development and communication and identity issues were particularly relevant. The parents’ group differed slightly from the other two groups, although decision making was especially challenging here, due to its vicarious nature. The diaries revealed, primarily, challenges that all three cochlear groups encountered in their daily lives, which mainly concerned technology (damage prevention, responsibility), social environment and communication. Overall, the patients felt that they could live a more self-determined life because of hearing through the implant. In the context of care, it was striking that patients felt they had to be firm and demanding to make their claims successfully.
**Glaucoma**	Holistic view of the disease Marbled experiences in the healthcare setting Fear of disease progression Adherence to drug therapy	In the group without a stent, participants agreed above all that there are deficits in the doctor–patient relationship, due to low level of sensitivity and empathy on the part of the medical profession. This was overcome mainly by the exchange of experiences within the group and exertion of personal responsibility in the care context and in the procurement of information. The diaries accentuated the importance of successfully integrating drop therapy in everyday life. Uncertainty regarding the progression of the disease was perceived as burdensome and resulted in constant self-monitoring as a coping strategy. All participants pleaded for a more holistic approach to manage glaucoma.
**Cardiovascular**	Confrontation with mortality No alternative Fear vs. security Life planning	Patients with cardiovascular implants felt confronted with their own mortality, which was reflected in the pronounced need for exchange with others, in part to deal with concomitant psychological stress. Participants also stated that this promoted their own engagement within the care setting. Subjective quality of life (in interaction with physical and emotional symptom burden) depended on the balance between uncertainty, anxiety, acceptance, and gratitude. The disease and its treatment seemed to have a strong impact on the personal sense of security and confidence in one’s own body, self-confidence and sense of normality of everyday life. The data showed that there was a difference regarding the perceived security and the acceptance of the implant between participants who were fitted with an implant out of an emergency situation and those who underwent a decision-making process regarding their implant.

**Table 6:** Individual and organisational HL development at a glance.

Patients’ Needs	Ethical Dimension	Future Research Topics	Practice
**(1) Information and perceptions regarding the implant, technology and disease**			
Improvement in health knowledge Comprehension of disease and risk factors Understanding actionability in terms of prevention	Empowerment in the field of decision making Responsibility of the system to provide understandable information and promote patients’ skills in handling it	Development and assessment of such interventions and use of stronger designs [61].	Interventions on understanding, comprehension, actionability, and satisfaction [3] that are tailored to the needs of patients, addressing functional, interactive and critical skills without using difficult animated spoken text [61].
Improvement in technological knowledge on implants function on functional range on surgery specifics	Empowerment in the field of decision making Information responsibility of the system	Relevance of technology-oriented HL in implant care and ways of integrating it in the healthcare system.	Interventions on informing, training and discussing technology-related themes.
Increasing awareness towards one’s own perceptions on technology and health	Empowerment—identity and decision making Value-oriented HL	Moral dimensions of HL and the impacts on individuals’ attitudes to decision making.	Interventions on increasing awareness for moral and ethical questions among affected individuals, their doctors and technicians.
**(2) Appraisal, dependence and responsibility**			
Increasing individuals’ tolerance of ambiguity	Empowerment in the field of decision making Information and communicative responsibility of the system	Factors that enable handling ambiguous technological and health information.	Interventions on an individual level—improving skills of handling ambiguous information—and on organisational level—offering paths and orientation frameworks.
Information needs on how to cope with psychological stress	Empowerment in the field of decision making Information responsibility of the system Value-oriented HL	Impact of psychological stress on decision making and the relation between technological risks and stress in the field of HL and health prevention.	Interventions for stress reduction in the context of implant care delivery and offering information and advice on coping strategies as well as possible supporting interventions (e.g., therapy, self-help groups, etc.).
Active involvement in health prevention and promotion	Empowerment Value-oriented HL	Effective ways of collecting and assessing fast-changing information on technical innovations and new therapies.	Interventions for increasing individual’s responsibility in terms of health prevention and promotion, providing holistic information on disease, and health and technology.
Supporting objective and subjective risk assessment	Empowerment Value-oriented HL	Factors that impact subjective risk assessment in the context of health and technology (especially in the context of technical and medical innovations).	Interventions that include the provision of resources (information, time, offer of discussion) by specialists with high communicative HL who can assist patients in the process of risk assessment and decision making.
Communicative skills both from providers and patients that enable individuals to interact with their doctors and extract and provide the necessary information through communication	Empowerment in the field of decision making Information responsibility of the system	Communicative action between doctors and patients.	Interventions for increasing individual HL through language (e.g., plain language), pedagogical techniques and clinical skills (e.g., shared decision making) [3].
**(3) Implant-related values**			
Skills of increasing the subjective quality of life	Empowerment Value-oriented HL Self-determination	Social, psychological and cultural aspects of implant development and implant care as well as the consequences for individuals after implantation.	Individual HL can be developed with interventions that, e.g., offer communication trainings or advice of how to deal with the new sense of hearing or a feeling of a foreign body in the eye, etc.
Ability to reconcile the individual values with the values of medicine and society	Empowerment Value-oriented HL Equity	Increasing the awareness of the moral dimension of individual and organisational HL in research may underline the importance of the co-construction of the concept using participatory approaches.	Interventions on HL development should raise awareness with regard to the major impact on identity, quality of life and life planning. From a moral perspective, organisations need to remove HL-related barriers that hinder “access to information, navigation of services, and decision making” [60].
Perceiving implant care as fair and affordable	Value-oriented HL Equity	Values of social justice and going beyond an individual and national cost benefit analysis [17].	Interventions on individual HL development should contain “meta-cognitive skills around critical thinking, self-awareness and citizenship rather than lists of practical skills” [17] and be open to revealing the power relations in their own framework (e.g., through intercultural comparisons, or case studies).

## Data Availability

Not applicable.

## Appendix A

**Table A1:** Group discussion: adapted to each implant type.

What Comes to Your Mind in Connection with the Implant and Medical Care When You Hear the Following Term?
Justice Quality of Life Safety Technology Self-determination Acceptance Damage prevention Patient well-being Health Powerlessness
Question Block 1: Previous history: decision for an implant, information, expectations, personal feelings
When and how did you first become aware of the possibility of receiving an implant?
Question Block 2: Present: How do you experience everyday life/your life with the implant?
How did you experience the counselling and care regarding your implant?
Question Block 3: Future: prospects, expectations, wishes, fears
Do you feel that your needs are adequately addressed in the health system? How do you see the future with regard to your life with an implant?

## Appendix B

**Table A2:** “Inspiration sheet” for the diaries: adapted to each implant type.

Diary Study “(Everyday) Life with an Implant” Inspirations for Your Diary	
What situations were relevant for you today in relation to your implant? Describe these situations! What did they trigger in you? When you think back to today, what thoughts about your implant were on your mind?	The implant
What feelings did you have about your implant today? How did you perceive yourself with your implant? Can you relate these feelings to a specific situation or trigger? How did you deal with these feelings and situations? What were you satisfied with and what would you perhaps like to do differently next time?	Emotions
In a certain situation, did your implant have an influence on how you planned or organised something in everyday life? What has changed for you in relation to it, i.e., improved or worsened? Has your quality of life today been influenced by the implant? How? Did you feel limited or restricted by the implant today, or did you feel supported in a particular way? In which situations?	Quality of Life
Did you have any questions, problems or concerns about the implant technique today? How did you address them?	Technology
Have you communicated with anyone today about the implant? With whom and why? Was there anything you would like to report in relation to it?	Care

## Appendix C

**Table A3:** Structure of the collective orientation patterns of GD and DS.

**Cochlea**	
**Group Discussion**	**Diary Study**
***Information and perceptions regarding the implant, technology and disease***	
*Perceptions on the implant as a physical object*	
Sensory perception; everyday life; implant as part of the person	Everyday life
*Perceptions on the implant’s functioning and damage prevention*	
Features and functions	Equipment and features; mobility and safety; planning and preparation; damage prevention
*Information and knowledge related to the implant and the disease*	
Differences between implant brands; fast development of technology; exchange of experiences; initiation to technology	Exchange of experiences; individual hearing story
***Appraisal, dependence and responsibility***	
*Appraisal of information and disease*	
Decision-making; attitudes towards technology	Decision-making; individual attitudes towards technology; physical and psychological effects; sense of hearing
*The role of the active patient*	
Proactive behaviour in care management	Navigation coping strategies
*Dependence on the healthcare system and the implants*	
Implant vs. other/future therapy options; (dependence on) technology; counselling and education; aggravated diagnosis of other diseases; quality of care	(dependence on) Technology; care; needs and wishes
*Attitudes, coping and responsibility*	
Implant education; implant handling	Coping strategies; implant handling; parental handling regarding children’s CI
***Implant related values***	
*Self-determination in the context of treatment irreversibility and perceptions of good life*	
Proxy decision-making for a child; autonomy in use of technology	Independence; limitations and challenges of the implant
*Identity and participation*	
(collective) Identity; participating in life (again); standing up for oneself; social dynamics and adaptation; competences	Activities and inclusion; support; non-hearing and the environment; reactions from the environment; sign language; CI wearer about CI in contact with others; restrictions and aids in communication
**Glaucoma**	
**Group Discussion**	**Diary Study**
**COP: *Information and perceptions regarding the implant, technology and disease***	
*Perceptions on the implant as a physical object*	
Implant and prevention	Implant perception
*Perceptions on the implant’s functioning and damage prevention*	
Implant and prevention	Functionality; influencing factors; vision/visual acuity
*Information and knowledge related to the implant and the disease*	
Information; little knowledge about the disease in the (medical) environment; early detection and diagnosis; inner ocular pressure (IOP)	Information and education; comorbidities; exchange and communication; family history
**COP: *Appraisal, dependence and responsibility***	
*Appraisal of information and disease*	
Quality of life and symptoms; decision-making; research and treatment options; holistic treatment approach	Experiencing glaucoma; accompanying symptoms
*The role of the active patient*	
Coordination of treatment and follow-up; assertiveness and HL; individual initiative (treatment and information)	Exchange and communication
*Dependence on the healthcare system and the implants*	
Doctor-patient relationship; education and instructions; drop therapy and measuring IOP; healthcare system and financing; quality of care	Operation, pre- and aftercare
*Attitudes, coping and responsibility*	
Drop therapy; stress; self-help groups; educating the surroundings	Handling symptoms; drop therapy and pressure control; aids; coping strategies
***COP: Implant related values***	
*Self-determination in the context of treatment irreversibility and perceptions of good life*	
Self-determination	Independence; quality of life
*Identity and participation*	
Self-determination	Personal environment
**Cardiovascular**	
**Group Discussion**	**Diary Study**
**COP: *Information and perceptions regarding the implant, technology and disease***	
*Perceptions on the implant as a physical object*	
Perceptions on the implant	Effects on everyday life (post-op)
*Perceptions on the implant’s functioning and damage prevention*	
Safety; functioning of the implant	Preventative action; heart problems and implant
*Information and knowledge related to the implant and the disease*	
Age and gender; education; implant type and innovation	Information gathering
**COP: *Appraisal, dependence and responsibility***	
*Appraisal of information and disease*	
Emergency situation or already known heart problems; decision-making	Medication; comorbidities
*The role of the active patient*	
Doctor-patient relationship; patient role	Patient role
*Dependence on the healthcare system and the implants*	
Quality of care; treatment/implantation; coordination of treatment and follow-up	Quality of care; additional treatments
*Attitudes, coping and responsibility*	
Psychological coping; educating; respect for own health	Coping strategies; anxiety and concerns
***COP: Implant related values***	
*Self-determination in the context of treatment irreversibility and perceptions of good life*	
Quality of life; gratitude	Attitudes towards the implant
*Identity and participation*	
Role of family; role of self-help groups; (collective) identity	Family and friends; burden vs. support for others; volunteering and engagement; effects on personality and self-image
